# Synthesis and Characterization of Superhydrophobic Epoxy Resin Coating with SiO_2_@CuO/HDTMS for Enhanced Self-Cleaning, Photocatalytic, and Corrosion-Resistant Properties

**DOI:** 10.3390/ma17081849

**Published:** 2024-04-17

**Authors:** Zhongmin Wang, Xiaoyu Zhou, Yongwei Shang, Bingkui Wang, Kecheng Lu, Weijiang Gan, Huajun Lai, Jiang Wang, Caimin Huang, Zongning Chen, Chenggang Hao, Enlang Feng, Jiacheng Li

**Affiliations:** 1 Guangxi Academy of Sciences, Nanning 530007, China; zmwang@guet.edu.cn (Z.W.); 19861822256@163.com (Y.S.); wangbingkui2024@163.com (B.W.); lkc203@163.com (K.L.); ganwj5@mail2.sysu.edu.cn (W.G.); laihuajun@gxas.cn (H.L.); haochenggang@gxas.cn (C.H.); 2 Guangxi Key Laboratory of Information Materials, Guilin University of Electronic Technology, Guilin 541004, China; zxy1115393086@163.com (X.Z.); waj124@guet.edu.cn (J.W.); luzhao_gx@guet.edu.cn (C.H.); 3 Key Laboratory of Solidification Control and Digital Preparation Technology (Liaoning Province), School of Materials Science and Engineering, Dalian University of Technology, Dalian 116024, China; znchen@dlut.edu.cn; 4 Geely Baikuang Group Co., Ltd., Baise 533000, China

**Keywords:** superhydrophobic, photocatalytic, self-cleaning, anti-corrosion

## Abstract

The exceptional corrosion resistance and combined physical and chemical self-cleaning capabilities of superhydrophobic photocatalytic coatings have sparked significant interest among researchers. In this paper, we propose an economical and eco-friendly superhydrophobic epoxy resin coating that incorporates SiO_2_@CuO/HDTMS nanoparticles modified with Hexadecyltrimethoxysilane (HDTMS). The application of superhydrophobic coatings effectively reduces the contact area between the metal surface and corrosive media, leading to a decreased corrosion rate. Additionally, the incorporation of nanomaterials, exemplified by SiO_2_@CuO core–shell nanoparticles, improves the adhesion and durability of the coatings on aluminum alloy substrates. Experimental data from Tafel curve analysis and electrochemical impedance spectroscopy (EIS) confirm the superior corrosion resistance of the superhydrophobic modified aluminum alloy surface compared to untreated surfaces. Estimations indicate a significant reduction in corrosion rate after superhydrophobic treatment. Furthermore, an optical absorption spectra analysis of the core–shell nanoparticles demonstrates their suitability for photocatalytic applications, showcasing their potential contribution to enhancing the overall performance of the coated surfaces. This research underscores the promising approach of combining superhydrophobic properties with photocatalytic capabilities to develop advanced surface modification techniques for enhanced corrosion resistance and functional properties in diverse industrial settings.

## 1. Introduction

Corrosion results in economic losses, safety incidents, environmental pollution, and other detrimental effects that require urgent attention in our society [1,2]. Typically, coating the substrates surface to isolate it from the surrounding environment is the most effective corrosion protection method [3,4].

Among various coatings, superhydrophobic materials exhibit a strong repellent effect towards liquids, thereby enabling oil/water separation [5], microplastics removal [6], photodegradation of dyes [7], desalination [8], heavy metal removal [9], corrosion resistance [10], and self-cleaning [11] capabilities. Generally, the wettability of a surface is influenced by its surface energy and roughness [12,13]. Therefore, exposure of a superhydrophobic surface to harsh environments (acidic or alkaline solutions, organic solvents, abrasion, washing, UV radiation, and high temperatures) can lead to the deterioration of surface roughness (loss of nanoparticles or substrate abrasion) or an increase in surface energy (decomposition of hydrophobic long chains), resulting in the loss of superhydrophobic properties and a significant reduction in the service life of superhydrophobic materials [14,15]. Constructing highly reliable hydrophobic and durable coatings remains a formidable challenge. To enhance the adhesion of superhydrophobic coatings effectively, the concept of “nanoparticles + binder” has been proposed to improve the durability of the coatings [16,17,18]. Surface-modified SiO_2_ nanoparticles, which are stable and inherently hydrophobic, are utilized in superhydrophobic coatings. Sharma et al. prepared a triethoxyoctylsilane-modified SiO_2_ nanoparticle-based superhydrophobic coating by the solution method to prevent the corrosion of mild steel [19]. Wang et al. reported a robust superhydrophobic SiO_2_/epoxy coating prepared by a one-step spraying method for corrosion protection of aluminum alloy [20]. Luque et al. constructed a spiky SiO_2_ nanoparticle supramolecular polymer superhydrophobic coatings applied to transparent oil–water separating [21].

Within the adhesive realm, epoxy resin emerges as a standout candidate due to its exceptional chemical stability, robust abrasion resistance, notable water repellency, and strong adhesion to substrates, positioning it as a favored alternative to fluoropolymers for formulating superhydrophobic coatings [22,23]. Despite these advantages, a common issue with many superhydrophobic surfaces is their inherent oleophilic nature, making them susceptible to contamination by organic pollutants and leading to a gradual decline in hydrophobicity and the eventual loss of self-cleaning properties [24,25]. Consequently, the fusion of superhydrophobicity with the photocatalytic decomposition of organic compounds has garnered considerable attention from the research community in recent years [26,27,28,29]. Semiconductor oxides such as TiO_2_ [30], SnO_2_ [31], ZnO [32], NiO [33], Cu_2_O [34], and CuO [35] are extensively utilized as photocatalysts in the degradation of various pollutants, encompassing dyes, organic contaminants, natural organic substances, and pharmaceutical compounds. Notably, copper oxide (CuO), featuring a band gap within the range of 1.2~2.1 eV, stands out as a critical *p*-type semiconductor renowned for its catalytic, optical, antimicrobial, and cost-efficient attributes [36,37,38]. Moreover, besides furnishing the requisite roughness for superhydrophobic coatings, SiO_2_ can function as a core material for incorporating additional functional elements, facilitating the creation of core–shell architectures. In the realm of photocatalysis, the interplay between distinct components in composite materials enhances the efficiency of separating photo-generated electron–hole pairs, thereby prolonging the lifespan of active electrons, holes, and radicals. Consequently, SiO_2_ is extensively employed as a carrier for semiconductor catalytic catalysts to fabricate core–shell configurations [39,40,41].

Herein, we present a straightforward approach for the fabrication of flower-shaped SiO_2_@CuO nanoparticles through liquid-phase reduction. A detailed analysis is conducted to elucidate the impact of physical structure and chemical composition on the performance of these nanoparticles. Subsequently, SiO_2_@CuO core–shell particles, surface-modified with HDTMS and dispersed in epoxy resin, were applied via spray coating onto an aluminum alloy substrate. This process aimed to develop long-lasting superhydrophobic coatings with the capability of photocatalytic degradation of organic compounds. The self-cleaning effectiveness and underlying principles of these coatings were thoroughly examined and discussed.

## 2. Materials and Methods

### 2.1. Materials

Tetraethyl orthosilicate (TEOS; reagent grade, 98%) was purchased from Shanghai Macklin Biochemical Technology Co., Ltd. (Shanghai, China). Polyethylene glycol (PEG; average Mn = 4000) was purchased from Shanghai Macklin Biochemical Technology Co., Ltd. (Shanghai, China). Trisodium citrate dihydrate (Na_3_Cit; analytically pure, 99%) was purchased from Tianjin Kemiou Chemical Reagent Co., Ltd. (Tianjin, China). Copper nitrate trihydrate (Cu(NO_3_)_2_·3H_2_O; analytically pure, 99%) was purchased from Beijing Yinuokai Technology Co., Ltd. (Beijing, China). Melamine (analytically pure, 99%) was purchased from Maya Reagents Co., Ltd. (Jiaxing, China). Hexadecyltrimethoxysilane (HDTMS; reagent grade, 98.85%) was purchased from Aladdin Reagents Co., Ltd. (Shanghai, China). Epoxy resin E-44 and curing agent (methyl hexahydrophthalic anhydride, reagent grade, 99%) were purchased from Guangzhou Als New Material Co., Ltd. (Guangzhou, China). Xylene (analytically pure, 99%) was purchased from Jinan Jiaxing Chemical Co., Ltd. (Jinan, China). Deionized water (DI water) was prepared in the laboratory using water-making equipment manufactured by Jinan AiKen Co., Ltd. (Jinan, China). The resistivity of DI water used in all experiments was ≥18 mΩ·cm^−1^.

### 2.2. Preparation Methods

**Figure 1:**
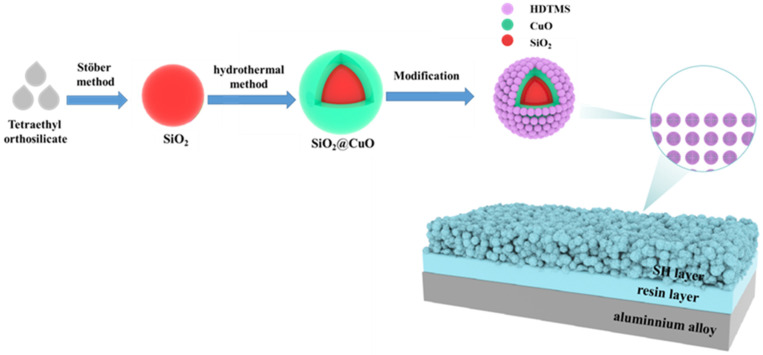
Schematic diagram of the preparation process of superhydrophobic coating.

The preparation method of superhydrophobic coating has four steps, and the process flow is shown in Figure 1.

Monodisperse spherical SiO_2_ particles were synthesized in a semi-batch reactor using sol-precipitation, which involved modifications to the Stöber method [42]. It begins by measuring 32.5 mL of absolute ethanol, 42.5 mL of deionized water, and 18 mL of ammonia water, and combining them in beaker A. Liquid A is prepared by magnetic stirring in a constant-temperature water bath set at 30 °C, with a stirring speed of 400 rpm. In beaker B, 8 mL of TEOS and 90 mL of absolute ethanol are measured and mixed to form solution B through magnetic stirring at 30 °C. The stirring speed of liquid A is then increased to 600 rpm, and within 1 min, liquid B is added to it. Subsequently, the stirring speed is reduced back to 400 rpm, and the reaction is maintained for two hours. After the mixed solution is obtained, it undergoes differential centrifugation once the ammonia water has evaporated. As a part of the process, a five-time wash with an alcohol–water mixture (*v*:*v* = 1:1) is employed to eliminate ammonia water and other impurities. The sample was subsequently dried in a constant temperature drying oven set at 60 °C for a duration of 3 h to achieve the monodisperse spherical SiO_2_ core.

In the second step, 0.001 mol of copper nitrate hydrate, 0.1 g of silicon dioxide, 0.1 g of polyethylene glycol, 0.2 g of melamine, and 12 mL of water are added to a container. The mixture is magnetically stirred at 500 rpm for 6 h in a water bath held at a constant temperature of 30 °C. Subsequently, 8 mL of 30% hydrogen peroxide is added, and stirring is continued for an additional 2 h. The mixture is then transferred to a hydrothermal kettle and allowed to react at 200 °C for 12 h. The solution is washed, and centrifugation is performed to collect the SiO_2_@CuO.

In the third step, 10 mL of water, 50 mL of ethanol, and 2 mL of HDTMS were individually added to the prepared silica and SiO_2_@CuO core–shell particles. The mixture was then reacted in a constant temperature water bath at 30 °C for 24 h. Subsequently, the centrifuged precipitate was collected, washed with ethanol, and dried at 60 °C for 3 h. Finally, the SiO_2_/HDTMS and SiO_2_@CuO/HDTMS modified with HDTMS are obtained.

In the fourth step, the aluminum alloy substrate (dimensions: 3 cm × 3 cm × 1 mm) underwent a series of preparation steps. Initially, it was meticulously cleaned using deionized water and subsequently subjected to a 10-min treatment with NaOH. This treatment induced chemical corrosion on the surface, thereby imparting roughness to the substrate. The treated substrate was subsequently washed with acetone and thoroughly dried in preparation for the resin coating. A mixture consisting of 0.1 g of epoxy resin, 0.3 g of curing agent, and 10 g of xylene was carefully combined, followed by sonication for 10 min to ensure homogeneity. The resulting mixture was then evenly sprayed onto the pre-treated substrate surface.

In the fifth step, 1 g of modified powder is combined with 10 g of xylene, subjected to ultrasonic dispersion for 10 min, and subsequently sprayed onto the aluminum alloy substrate using a spray gun. The resulting coating is baked at 140 °C for 2 h to achieve a superhydrophobic coating.

### 2.3. Characterization

The crystal phase of the sample was determined through X-ray diffraction (XRD) analysis in the 2θ range of 20–80°, using a Japanese Ultima IV instrument. Surface morphology was examined via scanning electron microscopy (SEM), employing a Czech TESCAN MIRA LMS instrument. The UV spectra of the samples were recorded using a Shimadzu UV-3600 spectrophotometer. The surface wettability was assessed by a contact angle meter system from GBX Scientific Instruments. Contact angle measurements were conducted to study the wettability. Methylene blue (MB) absorption was quantified using a Lambda EZ 210 UV–vis spectrophotometer. Electrochemical measurements were carried out utilizing a potentiostat/galvanostat/EIS setup from Ametek PARSTA 4000.

## 3. Results

### 3.1. Characterization of SiO_2_/CuO Core–Shell Particles

#### 3.1.1. Microstructure and Morphology

**Figure 2:**
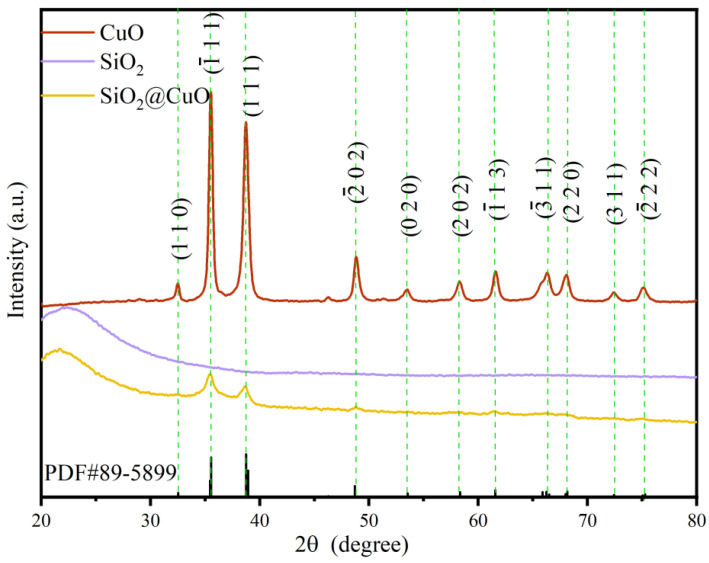
XRD patterns of CuO, SiO_2_, and SiO_2_@CuO.

Figure 2 presents the X-ray diffraction (XRD) results for CuO, SiO_2_, and SiO_2_@CuO. The SiO_2_ diffraction pattern displays a broad peak centered at 2θ = 21°, suggesting its amorphous nature. On the other hand, SiO_2_/CuO exhibits well-defined peaks at 2θ= 32.5°, 35.5°, 38.7°, 48.7°, 53.5°, 58.3°, 61.5°, 66.2°, 68.1°, 72.4°, and 75.2°, corresponding to the (110), ($\bar{1}$11), (111), ($\bar{2}$02), (020), (202), ($\bar{1}$13), ($\bar{3}$11), (220), (311), and ($\bar{2}$22) crystal planes of anatase SiO_2_. Additionally, in the XRD pattern of SiO_2_@CuO, a decrease in the CuO peak intensity indicates a reduction in CuO content.

**Figure 3:**
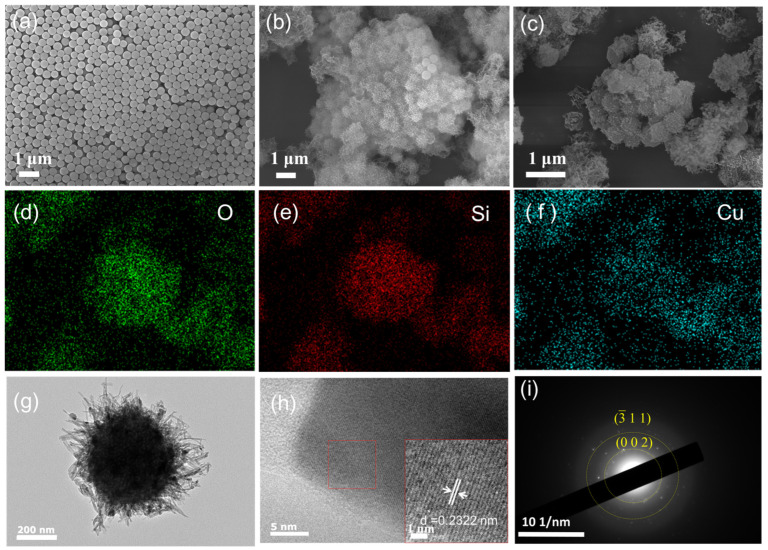
(**a**) SEM image of SiO_2_. (**b**,**c**) SEM images of SiO_2_@CuO. (**d**–**f**) Elemental distribution of oxygen (O), silicon (Si), and copper (Cu) pertaining to SiO_2_@CuO in (**c**). (**g**) TEM image of SiO_2_@CuO. (**h**) HRTEM image of SiO_2_@CuO. (**i**) SAED image analysis of selected regions of SiO_2_@CuO.

The micro/nano-hierarchical structure plays a critical role in the development of superhydrophobic surfaces. As depicted in Figure 3a, the SEM image of SiO_2_ showcases excellent dispersion and a smooth surface texture. Figure 3b presents the SEM image of the SiO_2_@CuO core–shell structure, formed through a standard hydrothermal reaction involving 0.1 g SiO_2_ and 0.001 mol Cu(NO_3_)_2_ hydrate. The images confirm the successful deposition of CuO onto the surface of SiO_2_, with CuO evenly distributed, leading to the formation of a micro/nanoscale hierarchical structure. Given that the micro/nano-hierarchical configuration is crucial for achieving superhydrophobic properties, the surface topography of the particles holds paramount significance.

The SEM image of SiO_2_@CuO core–shell particles post-calcination at 400 °C for 2 h is displayed in Figure 3c. Calcination is essential to remove any residual surfactants that might be present during the synthesis process, ensuring a clean and well-defined core–shell structure free from any disrupting surfactants [43]. Figure 3d–f depict the elemental mapping of oxygen (O), silicon (Si), and copper (Cu) within the structure exhibited in Figure 3c, revealing a close correlation between the distribution of these elements and the morphology of the SiO_2_@CuO core–shell particles. Figure 3g presents the TEM image of SiO_2_@CuO, demonstrating a relatively uniform loading of CuO onto the SiO_2_ surface to form the core–shell structure. The TEM images of SiO_2_@CuO/HDTMS nanoparticle was shown in Supplementary Figure S1. Compared with the SiO_2_@CuO, the shell layer of nanoparticles modified by HDTMS is damaged to a certain extent.

Figure 3h displays the HRTEM image of SiO_2_@CuO, while Figure 3i offers a closer look at Figure 3h, unveiling distinct crystal lattice patterns of CuO. The HRTEM image reveals an interplanar spacing of 0.2322 nm, aligning closely with the CuO (111) interplanar spacing (PDF#89-5899). Additionally, Figure 3i presents the selected area electron diffraction (SAED) image of SiO_2_@CuO, displaying the crystal planes (002) and ($\bar{3}$11) of CuO. Moreover, the morphological features of SiO_2_@CuO are consistent with observations from SEM analysis.

#### 3.1.2. Chemical Composition

**Figure 4:**
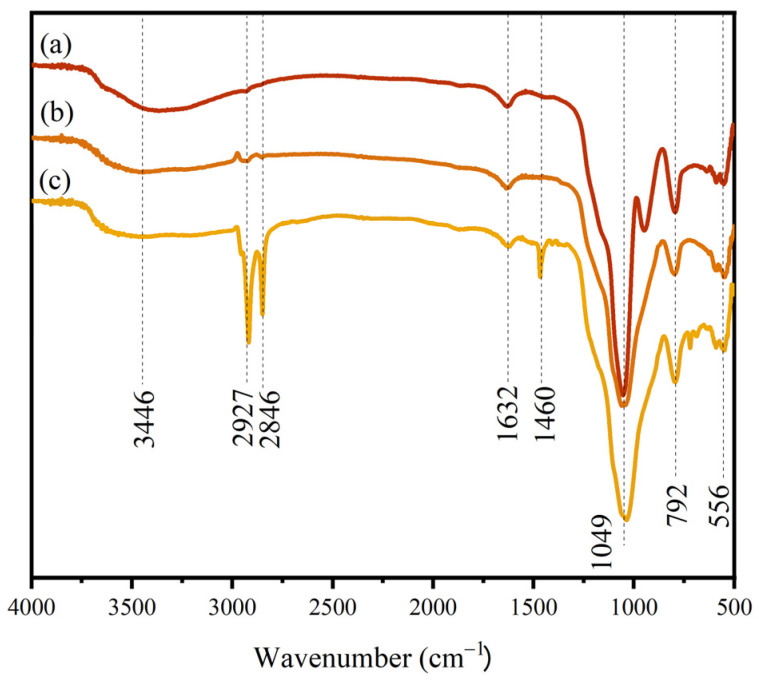
FT-IR spectra of (**a**) SiO_2_, (**b**) SiO_2_@CuO, and (**c**) SiO_2_@CuO/HDTMS.

The surface functional groups of the samples were analyzed using infrared spectroscopy. Figure 4a–c depict the FT-IR spectra of SiO_2_, SiO_2_@CuO, and SiO_2_@CuO/HDTMS, respectively. In all three samples, the broad peak observed at 3446 cm^−1^ corresponds to the –OH asymmetric absorption vibration peak of water, suggesting the presence of water adsorbed on their surfaces. The peak at 1049 cm^−1^ is associated with the asymmetric stretching vibration peak of Si–O–Si, while the peaks at 792 cm^−1^ and 556 cm^−1^ are related to the symmetric stretching and bending vibrations of Si–OH, respectively, signifying the characteristics of silica. In Figure 4c, which represents the spectrum of SiO_2_@CuO/HDTMS, the peaks at 2927 cm^−1^ and 2846 cm^−1^ are attributed to –CH_3_– and –CH_2_– groups. Additionally, the absorption peaks at 1632 cm^−1^ and 1460 cm^−1^ can be attributed to the stretching vibration peaks of the C–H bond. A comparison between Figure 4b,c reveals the presence of peaks related to –CH_3_–, –CH_2_–, and –CH, indicating the successful modification of SiO_2_@CuO particles with HDTMS. The results show that silane has successfully modified SiO_2_@CuO particles.

**Figure 5:**
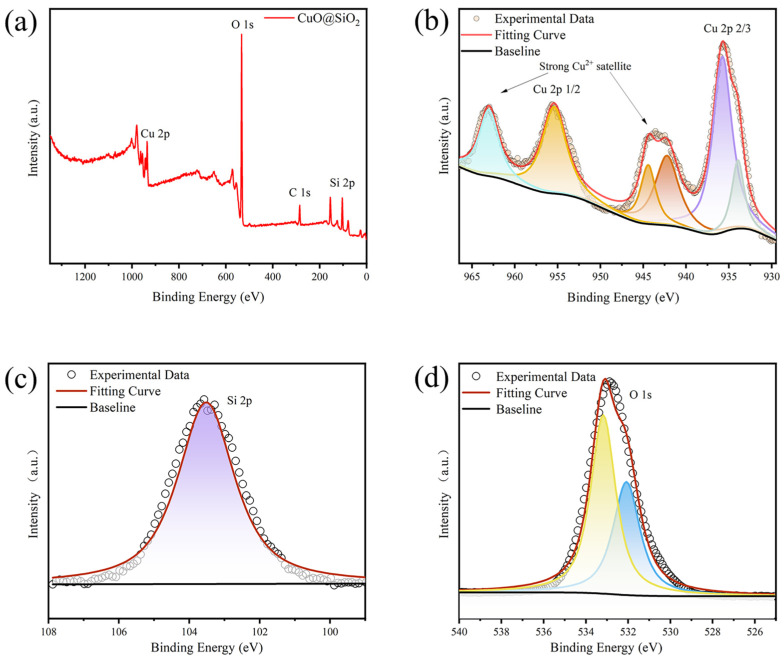
(**a**) XPS survey spectrum of the SiO_2_@CuO. XPS narrow scans spectra of (**b**) Cu 2p, (**c**) Si 2p, and (**d**) O 1s.

The X-Ray Photoelectron Spectroscopy (XPS) technique was employed to conduct a comprehensive analysis of the oxidation states and chemical composition of the synthesized SiO_2_@CuO core–shell particles. Figure 5a presents the full-scan spectrum from XPS, demonstrating the presence of Si, Cu, and O elements in the composite particles. The distinctive binding energies of Cu^2+^ and Cu^+^ verified the presence of Cu^2+^ in SiO_2_@CuO. The detailed XPS spectrum of Cu 2p in Figure 5b exhibits strong Cu^2+^ satellite peaks at binding energies of 942.5 eV and 962.5 eV, further confirming the existence of Cu^2+^ in SiO_2_@CuO. Moreover, a peak at 934.3 eV also supports the presence of Cu^2+^ in the composite material [44]. Additionally, a subtle peak at 932.1 eV was observed, indicating the presence of Cu species on the surface of SiO_2_@CuO. During the calcination process, the leftover organic material from the hydrothermal reaction facilitates the reduction of Cu^2+^. A single broad peak at 103.54 eV was identified in the Si 2p XPS spectrum, as shown in Figure 5c. This observation is consistent with the Si valence state of +4 in the SiO_2_ crystal phase [45]. Figure 5d displays the deconvoluted spectrum of the O 1s peak, which exhibits two distinct peaks. Previous studies suggest that the peak at a lower binding energy (532.0 eV) can be attributed to lattice oxygen species in metal oxides, while the peak at a higher binding energy (533.2 eV) is associated with oxygen in SiO_2_ and/or surface hydroxyl species on the SiO_2_@CuO system [46]. The presence of carbon in the spectra mainly arises from atmospheric contaminants. The deconvoluted C 1s spectra (Supplementary Figure S2) reveal three distinct binding energy values at 284.8 eV, 286.0 eV, and 288.5 eV, corresponding to the chemical bonds of C–C, C–N, and O–C=O, respectively [47].

#### 3.1.3. Bandgap Calculations

**Figure 6:**
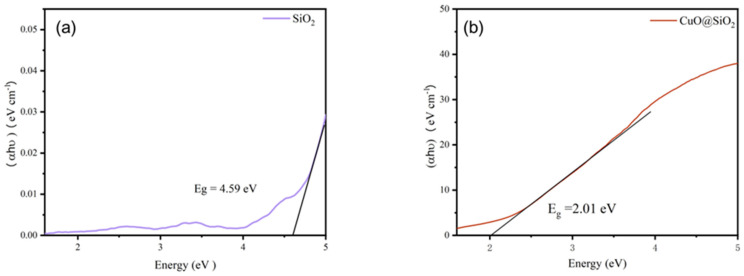
Optical absorption spectrum of (**a**) SiO_2_ and (**b**) SiO_2_@CuO.

By analyzing the optical absorption spectra of SiO_2_ and SiO_2_@CuO core–shell particles as shown in Figure 6, we can ascertain their band structure and edge characteristics. In the ultraviolet spectral range, a substantial increase in light absorption is observed, leading to the emergence of an absorption edge corresponding to the ultraviolet bandgap energy. Plotting the relationship between the absorption coefficient and photon energy of the SiO_2_@CuO core–shell particles revealed band gaps of 4.59 eV for SiO_2_ and 2.01 eV for SiO_2_@CuO. These data enable the determination that the wavelength of absorbable light is equal to or less than 616 nm.

### 3.2. Morphology of SiO_2_@CuO/HDTMS Coating

**Figure 7:**
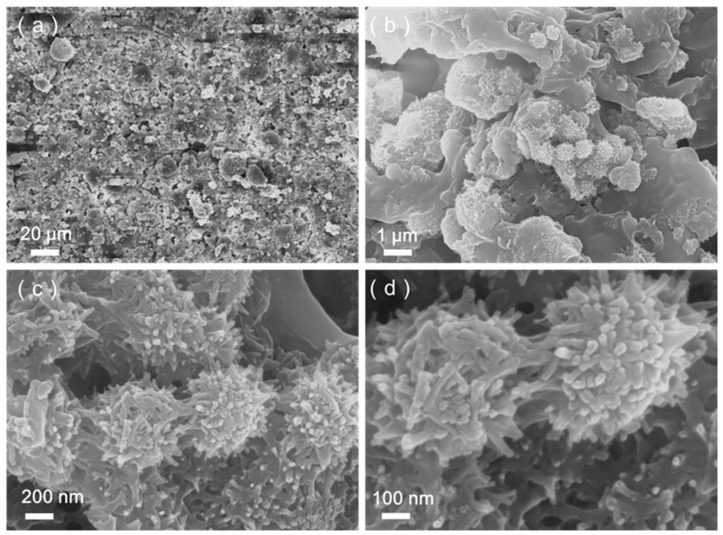
Surface morphology of the SiO_2_@CuO/HDTMS coating (**a**–**d**).

The surface morphology and thickness of the coating play a pivotal role in determining its wettability. Figure 7a–d depict the surface morphology of the SiO_2_@CuO/HDTMS coating. As illustrated in Figure 7a, the SiO_2_@CuO/HDTMS composite particles are uniformly dispersed on the coating surface, creating a rough surface structure. Figure 7b shows that some composite particles are partially exposed and bonded in place, effectively anchoring the hydrophobic particles. Figure 7c offers an enlarged view of Figure 7b, demonstrating the intact morphology and structure of the SiO_2_@CuO/HDTMS composite. Additionally, the micro/nanoscale layered rough structure on the coating’s surface acts as a foundational structure for achieving superhydrophobicity. Figure 7d presents a magnified image of Figure 7c. The study reveals that the morphology and structure of the SiO_2_@CuO composite remain unchanged after the modification and spraying processes, likely due to the formation of Si-O-Cu bonds between SiO_2_@CuO.

### 3.3. Liquid Repellent Properties of SiO_2_@CuO/HDTMS Coating

**Figure 8:**
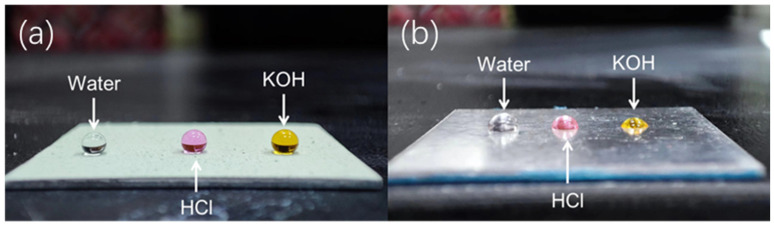
Liquid repellency of (**a**) the SiO_2_@CuO/HDTMS coating surface and (**b**) untreated Al slide surface towards water, HCl, and NaOH droplets.

Thorough investigation was carried out to evaluate the repellent properties of the prepared coatings and glass slides against water, acid droplets (pH = 2), and alkaline droplets (pH = 11). As shown in Figure 8a, droplets placed on the SiO_2_@CuO/HDTMS coating surface exhibit a spherical shape and demonstrate significant repellency to these liquids. In contrast, when the same droplets are placed on the surface of the aluminum alloy sheet, as shown in Figure 8b, they spread extensively, indicating the hydrophilic nature of the aluminum alloy sheet surface. Table 1 presents the Water Contact Angles (WCAs) of their surfaces for different droplets. The results unequivocally show that SiO_2_@CuO/HDTMS plays a critical role in conferring superhydrophobicity to the SiO_2_@CuO/HDTMS coatings. The hydrophobic functional groups of CH_2_=CH– and –CH_3_ in HDTMS are introduced into the surface of SiO_2_ and SiO_2_@CuO through modification, giving the material lower surface energy. Modified SiO_2_ and SiO_2_@CuO nanoparticles provided a nanoscale rough structure and lower surface energy after treatment. Nanoscale pits appeared between the materials. These pit structures can trap air, forming air pockets, causing fewer actual contact points between water and material, making it more difficult for water to spread on the surface. At this time, the contact state on the material is consistent with the Cassie model, and the water droplets easily roll off [48]. A comparison was conducted to assess the contact angle between our developed coating and the commercially available superhydrophobic coating (Ultra-ever Dry^®^) produced by Ultra Tech International (as shown in Supplementary Materials Table S1). The results demonstrate that the coating in our work exhibits a comparable contact angle to commercial materials, while its practical performance is enhanced by additional functionalities such as photocatalysis.

### 3.4. Self-Cleaning Property of SiO_2_@CuO/HDTMS Coating

**Figure 9:**
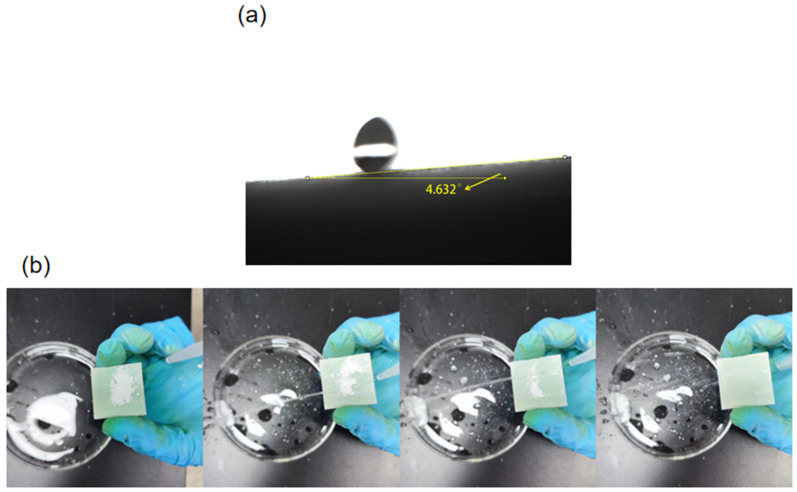
(**a**) The sliding angle (SA). (**b**) Simulation experiment of the self-cleaning of SiO_2_@CuO/HDTMS coating.

The sliding angle (SA) of the SiO_2_@CuO/HDTMS coating was measured with a value of 4.6° (Figure 9a). The high water-repellent property of the surface with low water-sliding angle helps to keep the surface clean like a lotus leaf. In the open air, many solid surfaces are contaminated by various types of dust particles. The self-cleaning efficacy of the coating was assessed through a simple simulation experiment, depicted in Figure 9b. In this setup, water droplets were deliberately dispensed from a syringe onto the coated surface. Remarkably, upon contact, the water droplets demonstrated an impressive self-cleaning capability, efficiently eliminating dirt and impurities from the surface. Consequently, the treated surface remained unsoiled and immaculate.

### 3.5. Durability of SiO_2_@CuO/HDTMS Coating

**Figure 10:**
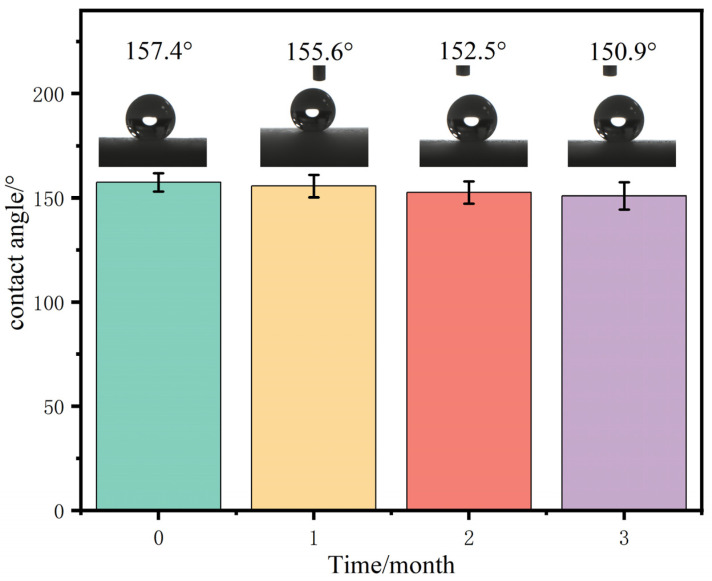
Durability of SiO_2_@CuO/HDTMS coating.

Coating stability is a critical property that must be thoroughly studied, particularly in outdoor environments. To assess the durability of the coatings, several samples were subjected to outdoor exposure for a three-month period. The changes in WCAs and their impact on photocatalytic degradation were analyzed. As shown in Figure 10, the initial WCA of the coating was 157.4°. Notably, after three months of exposure, there was minimal reduction in the WCA, with a measured value of 150.9° as compared to the initial reading.

### 3.6. Corrosion Resistance of the Coating

**Figure 11:**
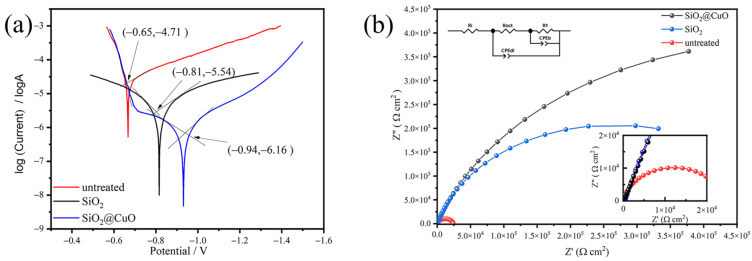
Tafel plots (**a**) and Nyquist plots (**b**) of the untreated aluminum alloy surface and superhydrophobic aluminum alloy surface in 3.5 wt% NaCl solution.

Corrosion resistance was evaluated by generating Tafel plots for both the untreated aluminum alloy surface and the superhydrophobic aluminum alloy surface, as shown in Figure 11a. Upon reaching a stable Open Circuit Potential (OCP) after immersion in a 3.5 wt% NaCl solution for at least one hour, the corrosion potential (*E*_corr_) and corrosion current density (*I*_corr_) were determined using the extrapolation method. The Ecorr and Icorr values for the untreated aluminum alloy surface presented in Table 1 were approximately −0.65 V and 1.9 × 10^−5^ A/cm^2^, respectively, while the superhydrophobic aluminum alloy surface showed values of −0.94 V and 6.9 × 10^−7^ A/cm^2^. A more positive Ecorr value indicates a lower risk of corrosion, while Icorr represents the corrosion rate. These results demonstrate that the superhydrophobic modification significantly reduces the likelihood and rate of corrosion on the aluminum alloy surface.

Electrochemical impedance spectroscopy (EIS) was employed for a comprehensive assessment of the corrosion performance. Equivalent circuit models were utilized to analyze the EIS data, offering detailed insights into the corrosion mechanisms. Figure 11b displays the EIS spectra obtained from the untreated aluminum alloy surface and the superhydrophobic aluminum alloy surface in the NaCl solution. The larger Nyquist curve associated with the superhydrophobic aluminum surface indicates a reduced corrosion rate compared to the conventional aluminum surface. This notable difference highlights a substantial enhancement in the corrosion resistance of the aluminum surface after the superhydrophobic treatment.

Based on the obtained *I*_corr_ (μA/cm^2^), the corrosion rate (CR) can be estimated using the following formula [49]:

(1) $$CR(mm/year)=\frac{\left(3.27\times 10^{-3}\times I_{corr}\times M\right)}{nd}$$

where *M* represents the metal’s relative atomic mass (in g/mol), *d* signifies the metal’s density (in g/cm^3^), and *n* represents the number of electrons required to oxidize an atom of the element during the corrosion process, corresponding to the metal’s valence state.

The Corrosion Inhibition Efficiency (CIE) can be calculated using the following formula [49]:

(2) $$CIE/\%=\frac{I_{corr}-I_{corr}^{\prime }}{I_{corr}}\times 100$$

where *I*_corr_ and *I*’_corr_ represent the corrosion current densities of the aluminum alloy surface before and after superhydrophobic modification, respectively. The computed values for the corrosion rate (CR) and Corrosion Inhibition Efficiency (CIE) are also presented in Table 2. These electrochemical parameters provide clear evidence that the superhydrophobic modification serves as an effective protective barrier for the aluminum surface, resulting in a remarkable 96.5% reduction in CR following the application of the superhydrophobic treatment. The presence of the entrapped air layer on the superhydrophobic surface restricts water contact with the interfacial matrix phase, thus effectively preventing corrosion ions from accessing the aluminum surface and corroding the metal barrier. Additionally, the presence of entrapped air within the layered micro/nanostructures creates a repelling effect on corrosion ions, attributed to the Laplace pressure [50]. The superhydrophobic modification noticeably delays the corrosion or degradation process of the aluminum surface.

### 3.7. Catalytic Degradation Performance

The photocatalytic performance of SiO_2_@CuO core–shell particles directly impact the SiO_2_@CuO/HDTMS coating. Figure 7 illustrates the results of diverse samples’ photocatalytic degradation performance assessments. During the initial dark reaction phase, the C/C_0_ values of various pollutant suspensions slightly decreased post-treatment with SiO_2_@CuO core–shell particles. 30 min later, the SiO_2_ group in the blank test reached adsorption equilibrium, indicating a certain level of pollutant adsorption by the samples. However, notable variations in degradation efficiency were observed among samples when exposed to different pollutant solutions in the photoreaction phase. After 300 min of UV irradiation, suspensions with SiO_2_ and the control group exhibited nearly identical C/C_0_ values, indicating that SiO_2_ lacked photocatalytic properties but retained some adsorption ability. In contrast, the SiO_2_@CuO composites showed effective photocatalytic performance. It is crucial to highlight that photolysis, conducted solely on the irradiated dye, helped evaluate the impact of UV light on photodegradation. A lower photolysis rate constant implies that degradation results from the photocatalyst rather than light exposure alone. The relatively lower photocatalytic degradation rate of CuO can be attributed to its narrow bandgap, causing rapid electron–hole pair recombination. When CuO interacts with photons exceeding its band gap energy, electron–hole pairs are generated. The resulting holes react with water molecules to yield hydroxyl radicals, while electrons convert dissolved oxygen into superoxide radicals. Protonation of superoxide ions leads to further hydroxyl radical formation. These highly reactive hydroxyl radicals, generated through light-assisted mechanisms, attack dye molecules, facilitating their degradation into water and carbon dioxide [51]. The superhydrophobic performance owing to the WCA and SA of the SiO2@CuO/HDTMS coatin developed in this work is superior to those observed for other similar coating, as shown in Supporting Materials Table S2.

**Figure 12:**
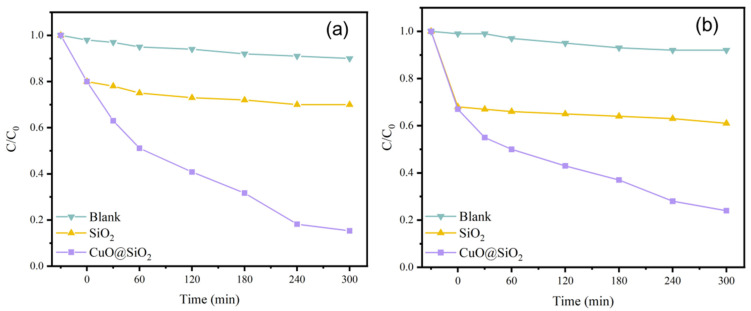
(**a**) Photocatalytic degradation effect of samples on rhodamine b. (**b**) Photocatalytic degradation effect of samples on methylene blue.

Figure 12a shows the 100 mg/L rhodamine b solution, and Figure 12b shows the 100 mg/L methylene blue solution. In the first 60 min, the SiO_2_@CuO composite particles decompose the two solutions at approximately the same rate. However, the subsequent rate of decomposition of rhodamine b by particles is higher than that of methylene blue, and the C/C_0_ value of rhodamine b after 300 min is 0.15, which is lower than that of methylene blue, 0.24.

### 3.8. Superhydrophobic and Photocatalytic Mechanism

**Figure 13:**
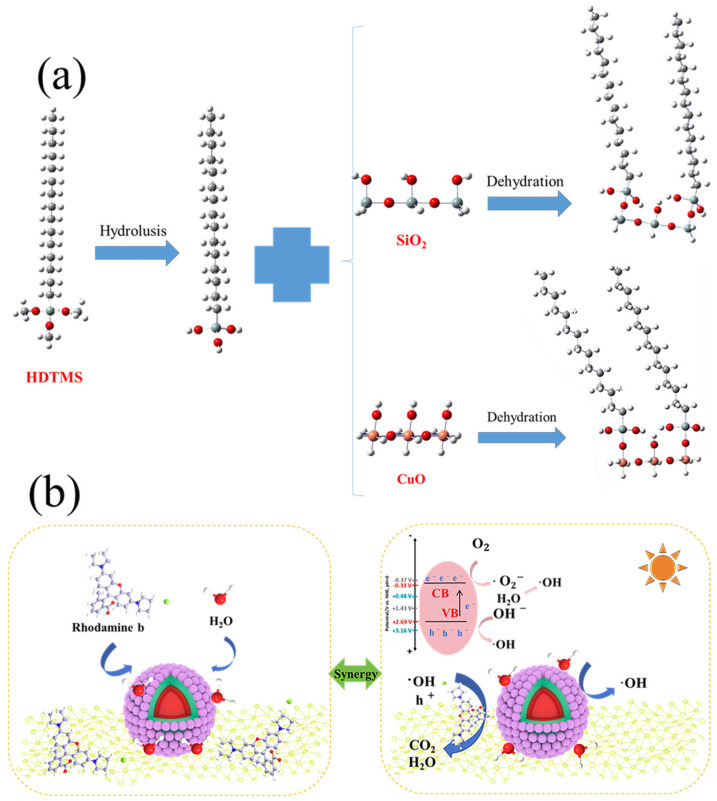
(**a**) Reaction mechanism of HDTMS and SiO_2_@CuO; (**b**) Schematic diagram of photocatalytic mechanism.

The variations in wettability and adhesion of the SiO_2_@CuO/HDTMS coating were analyzed by considering the obtained results alongside a theoretical model from the existing literature. In Figure 13a, the interaction process between HDTMS and SiO_2_@CuO is depicted. Both SiO_2_ and CuO exhibit hydrophilic characteristics. Upon hydrolysis of HDTMS, hexadecylsilanol containing an –OH group is produced. This –OH group then undergoes condensation with the –OH groups existing on the surfaces of SiO_2_ and CuO. Consequently, this process converts the SiO_2_@CuO core–shell particles into a superhydrophobic state post-HDTMS modification [52,53]. The findings indicate that the incorporation of Si–(CH_2_)_15_–CH_3_ onto the SiO_2_@CuO composite surface reduces the composite’s surface energy, thereby enhancing its superhydrophobic properties. The photocatalytic mechanism is detailed in Figure 13b. CuO, as a photosensitive material, produces electron-hole pairs when exposed to ultraviolet radiation. the accumulated e- in the conduction band (CB) of CuO, which possesses high potential energy, is capable of reducing oxygen to produce the superoxide radical •O^2−^. While the holes on the valence band (VB) of CuO can react with H_2_O to form •OH radicals. During the process of photo-oxidation, these free radicals efficiently degrade the dye, resulting in the production of harmless CO_2_ and H_2_O substances.

## 4. Conclusions

In summary, SiO_2_@CuO core–shell particles were synthesized using the hydrothermal method. The particles were then functionalized with HDTMS and sprayed onto an aluminum alloy surface, leading to the formation of a superhydrophobic coating with self-cleaning properties and photocatalytic functionalities. Through the application of superhydrophobic coatings, the contact area between the metal surface and corrosive media is minimized, leading to a reduction in the corrosion rate. The incorporation of nanomaterials, such as SiO_2_@CuO core–shell nanoparticles, further improves the adhesion and durability of superhydrophobic coatings on aluminum alloy substrates. Additionally, the utilization of copper oxide (CuO) nanoparticles for photocatalytic degradation of organic compounds demonstrates potential for enhancing the overall performance of the coated surfaces. The Tafel curve and EIS analysis showed a significant decrease in the corrosion rate of the aluminum alloy surface after superhydrophobic treatment, indicating an improved corrosion resistance compared to untreated surfaces. The combination of superhydrophobic coatings and photocatalytic degradation presents a promising approach to enhance the corrosion resistance of metal surfaces, particularly aluminum alloys.

## Figures and Tables

**Table 1:** WCAs of the surfaces of untreated Al slide and the SiO@CuO/HDTMS coating to different droplets.

Different Droplets	Water	HCI	NaOH
untreated Al	66.8 ± 1.2°	62.4 ± 1.5°	58.9 ± 2.3°
SiO_2_ @HDTMS coating	146.5 ± 3.4°	140.4 ± 4.1°	142.6 ± 3.9°
SiO_2_/CuO@HDTMS coating	157.4 ± 4.7°	152.5 ± 5.0°	153.4 ± 5.5°

**Table 2:** Corrosion potential (*E*_corr_), corrosion current density (*I*_corr_), CR, and the CIE of the untreated aluminum alloy surface and superhydrophobic aluminum alloy surface.

Sample	$E_{corr}$ (V)	$I_{corr}$ (A/cm^2^)	CR (mm/year)	CIE/%
untreated	−0.65	1.95 × 10^−5^	0.2124	0
SiO_2_	−0.81	2.88 × 10^−6^	0.0314	85.2
SiO_2_@CuO	−0.94	6.92 × 10^−7^	0.0075	96.5

## Data Availability

The data are available upon reasonable request from the corresponding author.

## Supplementary Materials

The following supporting information can be downloaded at https://www.mdpi.com/article/10.3390/ma17081849/s1, Figure S1: TEM images of SiO_2_@CuO/HDTMS nanoparticle; Figure S2: XPS narrow scans spectra of C1s; Figure S3: Before and after images of the degradation of (a) Rhodamine B and (b) Methylene Blue. The volumetric flask displays the color of the solution before degradation, while the centrifugal tube displays the color of the solution after degradation; Table S1: A comparison was conducted to assess the contact angle between our developed coating and the commercially available superhydrophobic coating (Ultra-ever Dry^®^) [54,55,56,57,58,59]; Table S2: Comparison of properties of self-cleaning surfaces through synergistic action of superhydrophobicity and photocatalytic activity [29,60,61,62,63,64,65,66,67,68].

## References

[1] Raabe D., Tasan C.C., Olivetti E.A. (2019). Strategies for improving the sustainability of structural metals. Nature.

[2] Xu D., Gu T., Lovley D.R. (2023). Microbially mediated metal corrosion. Nat. Rev. Microbiol..

[3] Salzano de Luna M. (2022). Recent Trends in Waterborne and Bio-Based Polyurethane Coatings for Corrosion Protection. Adv. Mater. Interfaces.

[4] Hooda A., Goyat M.S., Pandey J.K., Kumar A., Gupta R. (2020). A review on fundamentals, constraints and fabrication techniques of superhydrophobic coatings. Prog. Org. Coat..

[5] Thasma Subramanian B., Alla J.P., Essomba J.S., Nishter N.F. (2020). Non-fluorinated superhydrophobic spray coatings for oil-water separation applications: An eco-friendly approach. J. Clean. Prod..

[6] Al Harraq A., Bharti B. (2022). Microplastics through the lens of colloid science. ACS Environ. Au.

[7] Liu X., Wei Y., Tao F., Zhang X., Gai L., Liu L. (2022). All-water-based superhydrophobic coating with reversible wettability for oil-water separation and wastewater purification. Prog. Org. Coat..

[8] Xiao W., Yan J., Gao S., Huang X., Luo J., Wang L., Zhang S., Wu Z., Lai X., Gao J. (2022). Superhydrophobic MXene based fabric composite for high efficiency solar desalination. Desalination.

[9] Phiri I., Eum K.Y., Kim J.W., Choi W.S., Kim S.H., Ko J.M., Jung H. (2019). Simultaneous complementary oil-water separation and water desalination using functionalized woven glass fiber membranes. J. Ind. Eng. Chem..

[10] Gupta R., Verma R., Kango S., Constantin A., Kharia P., Saini R., Kudapa V.K., Mittal A., Prakash J., Chamoli P. (2023). A critical review on recent progress, open challenges, and applications of corrosion-resistant superhydrophobic coating. Mater. Today Commun..

[11] Dalawai S.P., Saad Aly M.A., Latthe S.S., Xing R., Sutar R.S., Nagappan S., Ha C.-S., Kumar Sadasivuni K., Liu S. (2020). Recent Advances in durability of superhydrophobic self-cleaning technology: A critical review. Prog. Org. Coat..

[12] Croll S.G. (2020). Surface roughness profile and its effect on coating adhesion and corrosion protection: A review. Prog. Org. Coat..

[13] Hashjin R.R., Ranjbar Z., Yari H., Momen G. (2022). Tuning up sol-gel process to achieve highly durable superhydrophobic coating. Surf. Interfaces.

[14] Zhang H., Guo Z. (2023). Recent advances in self-healing superhydrophobic coatings. Nano Today.

[15] Elhaddad F., Luna M., Gemelli G.M.C., Almoraima Gil M.L., Mosquera M.J. (2023). Effectiveness and durability assessment, under extreme environmental conditions, of a superhydrophobic coating applied onto sandstone from Carteia roman archaeological site. Chem. Eng. Sci..

[16] Zhou H., Chen R., Liu Q., Liu J., Yu J., Wang C., Zhang M., Liu P., Wang J. (2019). Fabrication of ZnO/epoxy resin superhydrophobic coating on AZ31 magnesium alloy. Chem. Eng. J..

[17] Syafiq A., Rahim N.A., Balakrishnan V., Pandey A.K. (2024). Development of self-cleaning polydimethylsiloxane/nano-calcium carbonate-titanium dioxide coating with fog-resistance response for building glass. Pigment. Resin Technol..

[18] Celik N., Torun I., Ruzi M., Esidir A., Onses M.S. (2020). Fabrication of robust superhydrophobic surfaces by one-step spray coating: Evaporation driven self-assembly of wax and nanoparticles into hierarchical structures. Chem. Eng. J..

[19] Sharma K., Malik M.K., Hooda A., Pandey K., Sharma J., Goyat M.S. (2023). Triethoxyoctylsilane-modified SiO_2_ nanoparticle-based superhydrophobic coating for corrosion resistance of mild steel. J. Mater. Eng. Perform..

[20] Zhang X.-F., Li X.-D., Wang N., Liu Y.-J., Tian F., Wang C.-X. (2023). Robust superhydrophobic SiO_2_/epoxy composite coating prepared by one-step spraying method for corrosion protection of aluminum alloy: Experimental and theoretical studies. Mater. Des..

[21] Nguyen N.B., Ly N.H., Tran H.N., Son S.J., Joo S.-W., Vasseghian Y., Osman S.M., Luque R. (2023). Transparent oil–water separating spiky SiO_2_ nanoparticle supramolecular polymer superhydrophobic coatings. Small Methods.

[22] Butt A.M., Wang Y., Ma H., Li H. (2023). The preparation of cerium nitrate and attapulgite based superhydrophobic epoxy coatings for the corrosion protection of Q355 mild steel surface. Surf. Coat. Technol..

[23] Ratnam D., Bhaumik S.K. (2024). Functionalized borosilicate-silica-epoxy nanocomposite superhydrophobic coating for corrosion inhibition under harsh environment. Prog. Org. Coat..

[24] Talinungsang, Upadhaya D., Kumar P., Purkayastha D.D. (2019). Superhydrophilicity of photocatalytic ZnO/SnO_2_ heterostructure for self-cleaning applications. J. Sol-Gel Sci. Technol..

[25] Bao Y., Chang J., Zhang Y., Chen L. (2022). Robust superhydrophobic coating with hollow SiO_2_/PAA-b-PS Janus microspheres for self-cleaning and oil–water separation. Chem. Eng. J..

[26] Azmoon P., Farhadian M., Pendashteh A., Tangestaninejad S. (2023). Adsorption and photocatalytic degradation of oilfield produced water by visible-light driven superhydrophobic composite of MIL-101(Cr)/Fe_3_O_4_-SiO_2_: Synthesis, characterization and optimization. Appl. Surf. Sci..

[27] Zhang W., Li S., Wei D., Zheng Z., Han Z., Liu Y. (2023). Fabrication of a fluorine-free photocatalytic superhydrophobic coating and its long-lasting anticorrosion and excellent antibacterial abilities. Prog. Org. Coat..

[28] Ahmad W., Ahmad N., Rasheed S., Nabeel M.I., Mohyuddin A., Riaz M.T., Hussain D. (2024). Silica-Based superhydrophobic and superoleophilic cotton fabric with enhanced self-cleaning properties for oil–water separation and methylene blue degradation. Langmuir.

[29] Ansari A., Nouri N.M. (2023). A one step self-cleaning surface with robust superhydrophobic and photocatalytic properties: Electrostatic sprayed fluorinated ethylene propylene mixed with nano TiO_2_–SiO_2_ particles. Ceram. Int..

[30] Meng A., Zhang L., Cheng B., Yu J. (2019). Dual Cocatalysts in TiO_2_ Photocatalysis. Adv. Mater..

[31] Roy H., Rahman T.U., Khan M.A.J.R., Al-Mamun M.R., Islam S.Z., Khaleque M.A., Hossain M.I., Khan M.Z.H., Islam M.S., Marwani H.M. (2023). Toxic dye removal, remediation, and mechanism with doped SnO_2_-based nanocomposite photocatalysts: A critical review. J. Water Process Eng..

[32] Dhull P., Sudhaik A., Raizada P., Thakur S., Nguyen V.-H., Van Le Q., Kumar N., Parwaz Khan A.A., Marwani H.M., Selvasembian R. (2023). An overview on ZnO-based sonophotocatalytic mitigation of aqueous phase pollutants. Chemosphere.

[33] Li Y., Li H., Li S., Li M., He P., Xiao Y., Chen J., Zhou Y., Ren T. (2024). Boosting the photocatalytic hydrogen evolution performance by fabricating the NiO/Zn_3_In_2_S_6_ p-n heterojunction. Appl. Surf. Sci..

[34] Budi S., Syafei D.I., Yusmaniar, Khasanah Q.F., Laxmianti D. (2022). Electrodeposition of Cu_2_O films at room temperature for methylene blue photodegradation. J. Phys. Conf. Ser..

[35] Rehman A., Aadil M., Zulfiqar S., Agboola P.O., Shakir I., Aly Aboud M.F., Haider S., Warsi M.F. (2021). Fabrication of binary metal doped CuO nanocatalyst and their application for the industrial effluents treatment. Ceram. Int..

[36] Madona J., Sridevi C., Velraj G., Dhayal Raj A., George A. (2023). Surfactant assisted morphology controlled CuO nanostructures for enhanced photocatalytic performance and bacterial growth inhibition. Mater. Sci. Eng. B.

[37] Shen R., Jiang C., Xiang Q., Xie J., Li X. (2019). Surface and interface engineering of hierarchical photocatalysts. Appl. Surf. Sci..

[38] Dursun S., Koyuncu S.N., Kaya İ.C., Kaya G.G., Kalem V., Akyildiz H. (2020). Production of CuO–WO_3_ hybrids and their dye removal capacity/performance from wastewater by adsorption/photocatalysis. J. Water Process Eng..

[39] Ullah S., Ferreira-Neto E.P., Pasa A.A., Alcântara C.C.J., Acuña J.J.S., Bilmes S.A., Martínez Ricci M.L., Landers R., Fermino T.Z., Rodrigues-Filho U.P. (2015). Enhanced photocatalytic properties of core@shell SiO_2_@TiO_2_ nanoparticles. Appl. Catal. B Environ..

[40] Khalid A., Ahmed R.M., Taha M., Soliman T.S. (2023). Fe_3_O_4_ nanoparticles and Fe_3_O_4_ @SiO_2_ core-shell: Synthesize, structural, morphological, linear, and nonlinear optical properties. J. Alloys Compd..

[41] Hou Z., Chu J., Liu C., Wang J., Li A., Lin T., François-Xavier C.P. (2021). High efficient photocatalytic reduction of nitrate to N_2_ by Core-shell Ag/SiO_2_@cTiO_2_ with synergistic effect of light scattering and surface plasmon resonance. Chem. Eng. J..

[42] Stöber W., Fink A., Bohn E. (1968). Controlled growth of monodisperse silica spheres in the micron size range. J. Colloid Interface Sci..

[43] Zare M., Moradi L. (2022). Preparation of hollow mesoporous boron nitride spheres with surface decorated by CuO: A bifunctional acid-base catalyst for the green synthesis of some heterocyclic [3,3,3] propellane derivatives in water media. Appl. Surf. Sci..

[44] Faheem M., Jiang X., Wang L., Shen J. (2018). Synthesis of Cu_2_O-CuFe_2_O_4_ microparticles from Fenton sludge and its application in the Fenton process: The key role of Cu_2_O in the catalytic degradation of phenol. RSC Adv..

[45] Zhan Z., Li Z., Yu Z., Singh S., Guo C. (2018). Superhydrophobic Al Surfaces with Properties of Anticorrosion and Reparability. ACS Omega.

[46] Yu G., Geng L., Wu S., Yan W., Liu G. (2015). Highly-efficient cocatalyst-free H2-evolution over silica-supported CdS nanoparticle photocatalysts under visible light. Chem. Commun..

[47] Geng L., Jian W., Jing P., Zhang W., Yan W., Bai F.-Q., Liu G. (2019). Crystal phase effect of iron oxides on the aerobic oxidative coupling of alcohols and amines under mild conditions: A combined experimental and theoretical study. J. Catal..

[48] Gong B., Ma L., Guan Q., Tan R., Wang C., Wang Z., Wang K., Liu C., Deng C., Song W. (2022). Preparation and particle size effects study of sustainable self-cleaning and durable silicon materials with superhydrophobic surface performance. J. Environ. Chem. Eng..

[49] Kumar M., Bhatt V., Nayal O.S., Sharma S., Kumar V., Thakur M.S., Kumar N., Bal R., Singh B., Sharma U. (2017). CuI nanoparticles as recyclable heterogeneous catalysts for C–N bond formation reactions. Catal. Sci. Technol..

[50] Xiang T., Han Y., Guo Z., Wang R., Zheng S., Li S., Li C., Dai X. (2018). Fabrication of Inherent Anticorrosion Superhydrophobic Surfaces on Metals. ACS Sustain. Chem. Eng..

[51] Bensouici F., Bououdina M., Dakhel A.A., Tala-Ighil R., Tounane M., Iratni A., Souier T., Liu S., Cai W. (2017). Optical, structural and photocatalysis properties of Cu-doped TiO_2_ thin films. Appl. Surf. Sci..

[52] Cai Y., Zhao Q., Quan X., Feng W., Wang Q. (2020). Fluorine-free and hydrophobic hexadecyltrimethoxysilane-TiO_2_ coated mesh for gravity-driven oil/water separation. Colloids Surf. A Physicochem. Eng. Asp..

[53] Shirke Y.M., Yu Y.J., Chung J.-W., Cho S.-J., Kwon S.J., Hong S.U., Jeon J.-D. (2024). Hydrophobic hollow fiber composite membranes based on hexadecyl-modified SiO_2_ nanoparticles for toluene separation. J. Environ. Chem. Eng..

[54] Wang L., Yang J., Zhu Y., Li Z., Sheng T., Hu Y.M., Yang D.-Q. (2016). A study of the mechanical and chemical durability of Ultra-Ever Dry Superhydrophobic coating on low carbon steel surface. Colloids Surf. A.

[55] Hermann M., Agrawal P., Koch I., Oleschuk R. (2019). Organic-free, versatile sessile droplet microfluidic device for chemical separation using an aqueous two-phase system. Lab Chip.

[56] Oomen P.E., Mulder J.P.S.H., Verpoorte E., Oleschuk R.D. (2017). Controlled, synchronized actuation of microdroplets by gravity in a superhydrophobic, 3D-printed device. Anal. Chim. Acta.

[57] Hu W.-H., Yang D.-Q., Sacher E. (2018). Improving the Mechanical Durability of Superhydrophobic Coating by Deposition onto a Mesh Structure. Mater. Res. Express.

[58] Salahuddin M., Uddin M.N., Hwang G., Asmatulu R. (2018). Superhydrophobic PAN nanofibers for gas diffusion layers of proton exchange membrane fuel cells for cathodic water management. Int. J. Hydrogen Energy.

[59] Samah W., Clain P., Rioual F., Fournaison L., Delahaye A. (2023). Experimental investigation on the wetting behavior of a superhydrophobic surface under controlled temperature and humidity. Colloids Surf. A.

[60] Zhu H., Huang Y., Zhang S.-W. (2021). A universal, multifunctional, high-practicability superhydrophobic paint for waterproofing grass houses. NPG Asia Mater..

[61] Zhao S., Yang X.-T., Xu Y.-Y. (2022). A sprayable superhydrophobic dental protectant with photo-responsive anti-bacterial, acid-resistant, and anti-fouling functions. Nano Res..

[62] Keshavarzi R., Molabahrami N., Afzali N. (2020). Improving Efficiency and Stability of Carbon-Based Perovskite Solar Cells by a Multifunctional Triple-Layer System: Antireflective, UV-Protective, Superhydrophobic, and Self-Cleaning. Sol. RRL.

[63] Wang T., Lu Z.-C., Wang X.-Q. (2021). A compound of ZnO/PDMS with photocatalytic, self-cleaning and antibacterial properties prepared via two-step method. Appl. Surf. Sci..

[64] Bai X., Yang S.-Q., Tan C.-H. (2022). Synthesis of TiO_2_ based superhydrophobic coatings for efficient anti-corrosion and self-cleaning on stone building surface. J. Clean. Prod..

[65] Rahman A., Rahman A. (2022). Silver Oxide-Decorated Silica Nanoparticles for Visible-Light-Driven Photolytic Pollutant Degradation and Water-Oil Separation. ACS Appl. Nano Mater..

[66] Trávníčková E., Pijáková B., Marešová D. (2020). Antifouling performance of photocatalytic superhydrophobic coatings against Klebsormidium alga. J. Environ. Chem. Eng..

[67] Zhang M., Zhao M.-W., Chen R.-R. (2020). Fabrication of the pod-like KCC-1/TiO_2_ superhydrophobic surface on AZ31 Mg alloy with stability and photocatalytic property. Appl. Surf. Sci..

[68] Yang M., Jiang C., Liu W.-Q. (2020). A water-rich system of constructing durable and fluorine-free superhydrophobic surfaces for oil/water separation. Appl. Surf. Sci..

