# Supplementary material for: Synthesis and Characterization of Superhydrophobic Epoxy Resin Coating with SiO2@CuO/HDTMS for Enhanced Self-Cleaning, Photocatalytic, and Corrosion-Resistant Properties
Source: Materials (Basel). 2024 Apr 17;17(8):1849. doi: 10.3390/ma17081849 (PMC11051187; doi:10.3390/ma17081849)
Supplement: Supplementary file 1 [file materials-17-01849-s001.zip › materials-2934241-supplementary.pdf]

Supporting Information

Synthesis and Characterization of Superhydrophobic Epoxy Resin Coating with SiO_2@CuO/HDTMS for Enhanced Self-Cleaning, Photocatalytic, and Corrosion-Resistant Properties

Zhongmin Wang, Xiaoyu Zhou, Yongwei Shang, Bingkui Wang, Kecheng Lu, Weijiang Gan, Huajun Lai, Jiang Wang, Caimin Huang, Zongning Chen, Chenggang Hao, Enlang Feng and Jiacheng Li

Table S1. A comparison was conducted to assess the contact angle between our developed coating and the commercially available superhydrophobic coating (Ultra-ever Dry®).

No.	Substrate	Coating	Superhydrophobicity	Ref.
1	Stainless steel	Ultra-ever Dry®	WCA = 155.0°	[1]
2	Glass slides	Ultra-ever Dry®	WCA = 151.2°	[2]
3	Glass slides	Ultra-ever Dry®	WCA = 152.6°	[3]
4	Nylon mesh	Ultra-ever Dry®	WCA = 151.3°	[4]
5	Carbon fiber	Ultra-ever Dry®	WCA = 161.2°	[5]
6	Aluminum alloy	Ultra-ever Dry®	WCA = 157.6°	[6]
7	Aluminum alloy	SiO_2@CuO/HDTMS	WCA = 157.4°	This work

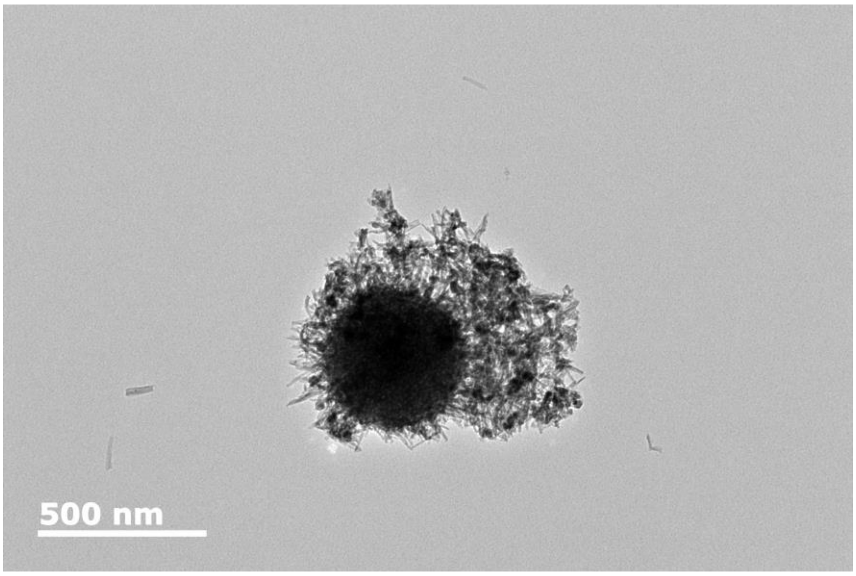

Figure S1. TEM images of SiO_2@CuO/HDTMS nanoparticle.

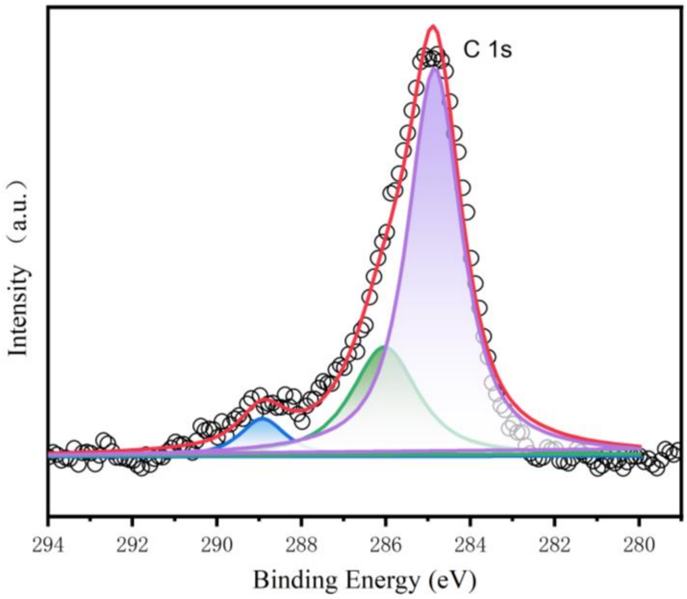

Figure S2. XPS narrow scans spectra of C1s.

Commented [M1]: Please check if there should be explanation for the colorful lines in the figure. Case of yes, please provide

Table S2. Comparison of properties of self-cleaning surfaces through synergistic action of superhydrophobicity and photocatalytic activity.

Materials	Super-hydrophobicity	Photocatalytic activity	Applications	Ref.
Dual-scale TiO_2/ ER/FAS	WCA >150° θSA < 10°	0.6893 h^{-1} for NR degradation under UV light	Self-cleaning Anti-icing	Zhu et al. (2021) [7]
FAS-SiO2/ZnO/ PDMS	WCA = 151° θ_{SA} = 2°	0.0073 h^{-1} for RhB degradation under yellow light	Self-cleaning Antifouling against stains Antibacterial	Zhao et al. (2022) [8]
TMCS-SiO_2/TiO_2/ FEP	WCA >150° $\theta_{SA} < 3_{o}$	0.6931 h^{-1} for MB degradation under UV light	Self-cleaning Antifouling against algae	Ansari and Nouri (2023) [9]
CeO_2/SiO_2/TiO_2/ PDMS	WCA = 153.4°	NA for OA degradation under UV light	Self-cleaning UV-blocking Anti-reflection	Keshavarzi et al. (2020) [10]
ZnO nanorods/ PDMS	WCA = 158.1°	1.2166 h^{-1} for MB degradation under UV light	Self-Cleaning Antibacterial	Wang et al. (2021b) [11]

TMCS-TiO_2@SiO_2/ Paraloid B72	WCA = ~150°	5.7420 h^{-1} for MB degradation under UV light	Self-cleaning Anti-corrosion	Bai et al. (2022) [12]
Ag_2O/SiO_2/TMCS	WCA >150° θ_{SA} < 8°	NA for RhB degradation under visible light	Self-cleaning Oil/water separation	(Rahman and R, 2022) [13]
TiO_2/UltraEverDry	WCA = 152.9°	0.1815 h^{-1} for NO degradation under UV light 0.0192 h^{-1} for NO degradation under	Self-cleaning Antifouling against algae	Trávníčková et al. [14]
TiO_2/wrinkled-SiO_2	WCA = 156° θ_{SA} = 6.6°	0.0854 h^{-1} for MO degradation under UV light	Self-cleaning Antifouling against stains Anti-corrosion	Zhang et al. (2020) [15]
TiO_2/PDMS	WCA = 156.9° θ_{SA} = 6.8°	NA for OR/MB degradation under UV light	Self-cleaning Oil/water separation Water Purification	Yang et al. (2020a) [16]
SiO_2@CuO/HDTMS	WCA = 157.4° θ_{SA} = 4.6°	0.5257 h^{-1} for RhB degradation under UV light	Self-cleaning Anti-corrosion	This work

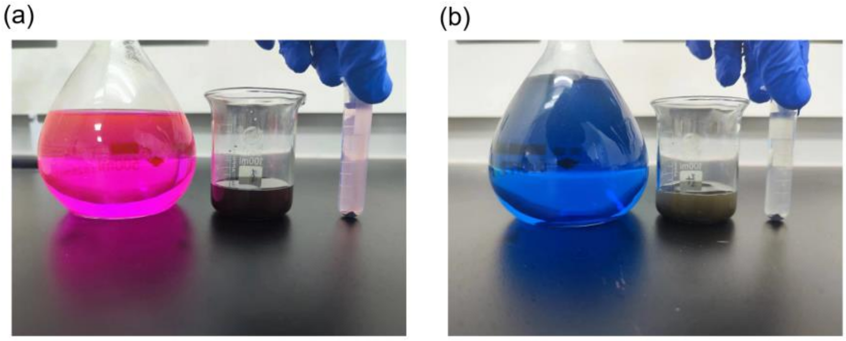

Figure S3. Before and after images of the degradation of (a) Rhodamine B and (b) Methylene Blue. The volumetric flask displays the color of the solution before degradation, while the centrifugal tube displays the color of the solution after degradation.

1. Wang, L.; Yang, J.; Zhu, Y.; Li, Z.; Sheng, T.; Hu, Y. M.; Yang, D.-Q., A study of the mechanical and chemical durability of Ultra-Ever Dry Superhydrophobic coating on low carbon steel surface. *Colloids and Surfaces A: Physicochemical and Engineering Aspects* **2016,** 497, 16-27.
2. Hermann, M.; Agrawal, P.; Koch, I.; Oleschuk, R., Organic-free, versatile sessile droplet microfluidic device for chemical separation using an aqueous two-phase system. *Lab on a Chip* **2019,** 19, (4), 654-664.
3. Oomen, P. E.; Mulder, J. P. S. H.; Verpoorte, E.; Oleschuk, R. D., Controlled, synchronized actuation of microdroplets by gravity in a superhydrophobic, 3D-printed device. *Analytica Chimica Acta* **2017,** 988, 50-57.
4. Hu, W.-H.; Yang, D.-Q.; Sacher, E., Improving the Mechanical Durability of Superhydrophobic Coating by Deposition onto a Mesh Structure. *Materials Research Express* **2018,** 5, (6), 065521.
5. Salahuddin, M.; Uddin, M. N.; Hwang, G.; Asmatulu, R., Superhydrophobic PAN nanofibers for gas diffusion layers of proton exchange membrane fuel cells for cathodic water management. *International Journal of Hydrogen Energy* **2018,** 43, (25), 11530-11538.
6. Samah, W.; Clain, P.; Rioual, F.; Fournaison, L.; Delahaye, A., Experimental investigation on the wetting behavior of a superhydrophobic surface under controlled temperature and humidity. *Colloids and Surfaces A: Physicochemical and Engineering Aspects* **2023,** 656, 130451.
7. Zhu, H.; Huang, Y.;Zhang, S.-W., A universal, multifunctional, high-practicability superhydrophobic paint for waterproofing grass houses. *NPG Asia Materials* **2021**. 13(1),47.
8. Zhao, S.; Yang, X.-T.; Xu, Y.-Y., A sprayable superhydrophobic dental protectant with photo-responsive anti-bacterial, acid-resistant, and anti-fouling functions. *Nano Research* **2022**. 15(6),5245-5255.
9. Ansari, A. and N.M. Nouri, A one step self-cleaning surface with robust superhydrophobic and photocatalytic properties: Electrostatic sprayed fluorinated ethylene propylene mixed with nano TiO2-SiO2 particles. *Ceramics International* **2023**. 49(1), 57-66.
10. Keshavarzi, R.; Molabahrami, N.; Afzali, N., Improving Efficiency and Stability of Carbon-Based Perovskite Solar Cells by a Multifunctional Triple-Layer System: Antireflective, UV-Protective, Superhydrophobic, and Self-Cleaning. *Solar RRL* **2020**. 4(12),2000491.
11. Wang, T., Lu, Z.-C.; Wang, X.-Q., A compound of ZnO/PDMS with photocatalytic, self-cleaning and antibacterial properties prepared via two-step method. *Applied Surface Science* **2021**. 550, 149286.
12. Bai, X., Yang,S.-Q.;Tan, C.-H., Synthesis of TiO2 based superhydrophobic coatings for efficient anti-corrosion and self-cleaning on stone building surface. *Journal of Cleaner Production* **2022**. 380,134975.

13 Rahman,A. ;Rahman A.,Silver Oxide-Decorated Silica Nanoparticles for Visible-Light-Driven Photolytic Pollutant Degradation and Water-Oil Separation. *ACS Applied Nano Materials* **2022**. 5(1),939-947.

14. Trávníčková, E.; Pijáková, B.; Marešová, D., Antifouling performance of photocatalytic superhydrophobic coatings against Klebsormidium alga. *Journal of Environmental Chemical Engineering* **2020**. 8(5),104153.

15. Zhang, M.; Zhao, M.-W.; Chen, R.-R., Fabrication of the pod-like KCC-1/TiO2 superhydrophobic surface on AZ31 Mg alloy with stability and photocatalytic property. *Applied Surface Science* **2020**. 499,143933.

16. Yang, M.; Jiang, C.; Liu, W.-Q., A water-rich system of constructing durable and fluorine-free superhydrophobic surfaces for oil/water separation. *Applied Surface Science* **2020**. 507,145165.
